# Telemedicine education for medical students and residents worldwide: A scoping review protocol

**DOI:** 10.1371/journal.pone.0359776

**Published:** 2026-10-01

**Authors:** Joshua Vo, Tina Mai, Nhi Yen Bui, Dang Ngoc Khoi, Minh Phan Ngoc Nguyen, Quang Thanh Nguyen

**Affiliations:** 1 Virginia Commonwealth University School of Medicine, Richmond, Virginia, United States of America; 2 University of California, San Francisco School of Medicine, San Francisco, California, United States of America; 3 College of Health Sciences, VinUniversity, Gia Lam, Hanoi, Vietnam; 4 Department of Surgery, Vietnam National Children’s Hospital, Dong Da, Hanoi, Vietnam; 5 The Royal College of Surgeons in Ireland and University College Dublin Malaysia Campus, George Town, Penang, Malaysia; 6 Innovations in Health Sciences, VinUniversity, Gia Lam, Hanoi, Vietnam; Shiraz University of Medical Sciences, IRAN, ISLAMIC REPUBLIC OF

## Abstract

Telemedicine has become an increasingly important component of contemporary health care delivery, yet its integration into undergraduate and graduate medical education remains variable and insufficiently standardized. Although individual studies have described telemedicine training initiatives, the literature remains fragmented across disciplines, learner groups, and geographic settings, limiting understanding of how telemedicine is taught, learned, assessed, and implemented for medical trainees worldwide. This scoping review aims to map the characteristics of telemedicine education for medical students enrolled in undergraduate or graduate entry physician training programs and for resident physicians, examine variation across learner groups, specialties, and geographic contexts, and identify reported implementation factors, barriers, facilitators, and evidence gaps. The review will follow Joanna Briggs Institute methodology for scoping reviews. A comprehensive search will be conducted in MEDLINE, Embase, the Cochrane Central Register of Controlled Trials, Education Resources Information Center, Web of Science, and the Cumulative Index to Nursing and Allied Health Literature, with additional studies identified through reference list screening of included articles. Two reviewers will independently screen titles, abstracts, and full-texts against predefined eligibility criteria and will extract data using a structured, piloted form. Findings will be synthesized descriptively in tabular and narrative formats to characterize curricular content, teaching methods, assessment strategies, and contextual influences on implementation. By providing a comprehensive map of the existing literature, this review will inform future curriculum development, educational research, and policy related to telemedicine training in medical education. **Review registration:** Open Science Framework (OSF) (https://osf.io/jxudv).

## Introduction

The rapid expansion of digital health technologies has transformed the delivery of healthcare worldwide, with telemedicine emerging as a central component of modern clinical practice [1,2]. Accelerated by the COVID-19 pandemic, telemedicine has shifted from a supplementary tool to a core modality for patient care across diverse settings [2–5]. As health systems increasingly integrate virtual care, there is a growing expectation that physicians and, by extension, medical trainees develop competencies in telemedicine, including remote clinical assessment, digital communication, and technology-enabled decision-making [6]. Despite this shift, the incorporation of telemedicine into formal medical education remains variable and often lacks standardization [7–9].

Within the last decade, medical schools and residency programs have begun to introduce telemedicine curricula to address these evolving demands [9,10]. These curricula encompass training on a range of virtual modalities, including didactic lectures, simulation-based training, e-learning platforms, case-based learning, and supervised virtual clinical encounters [8]. Similarly, assessment strategies have expanded to include written examinations, Objective Structured Clinical Examinations (OSCEs), and workplace-based assessments adapted for virtual care contexts [11–13]. However, the structure, depth, and implementation of telemedicine education differ widely across institutions and regions, reflecting variations in resources, technological infrastructure, regulatory environments, and educational priorities.

Beyond curricular design, the implementation and sustainability of telemedicine education are influenced by multiple contextual factors. These include institutional readiness, faculty expertise, access to digital platforms, and broader health system integration of telemedicine services [8,9]. As telemedicine curricula remain in relatively early stages of adoption, traditional models of instruction, where learners are trained primarily by established experts, may be insufficient [8,9]. Instead, institutions may need to support concurrent development of both faculty and trainees, with educators themselves requiring training in telemedicine competencies alongside learners [13,14]. In addition, learners’ attitudes, confidence, and perceptions toward telemedicine play a critical role in shaping engagement and skill acquisition, while educators may face challenges related to training, evaluation, and adapting conventional teaching approaches to virtual care contexts [13,15]. Together, these factors contribute to substantial heterogeneity in how telemedicine competencies are taught, assessed, and reinforced.

Existing studies and reviews have described individual telemedicine curricula, competency frameworks, learner outcomes, and implementation experiences [13,16–18]. However, to our knowledge, no comprehensive synthesis has mapped these elements together for both medical students and resident physicians across specialties, healthcare systems, and diverse educational and geographic settings. This gap is increasingly important because the rapid expansion of virtual care may outpace the development of coherent educational standards, leaving institutions to design curricula without a consolidated evidence base.

Accordingly, this scoping review will map curricular structures, competency domains, teaching strategies, assessment methods, and implementation conditions across undergraduate and postgraduate medical education. By identifying common practices, contextual variation, and evidence gaps, the review may provide an empirical foundation for future consensus-building, curriculum standards, faculty development, assessment design, and context-sensitive implementation guidance.

### Review questions

This review aims to map the nature, variation, and implementation of telemedicine education for physician trainees, including medical students enrolled in undergraduate or graduate entry medical degree programs and resident physicians globally:

1. What are the characteristics of telemedicine education for medical students and resident physicians, including curricular content, teaching methods, and assessment strategies?
2. How does telemedicine education vary across learner groups, specialties, and geographic contexts?
3. What implementation factors, barriers, facilitators, and evidence gaps are reported in telemedicine education for medical students and resident physicians?

## Methods

### Scoping-review rationale and framework

A scoping review was selected because telemedicine education is a broad, heterogeneous, and rapidly developing field. The literature spans multiple learner groups, specialties, teaching modalities, assessment approaches, and implementation settings, making a scoping review the most appropriate method for mapping the range and nature of the available evidence [19]. Scoping reviews are particularly useful for clarifying key concepts, examining how research in an area has been conducted, and identifying gaps in knowledge [19]. They also enable the inclusion of diverse forms of evidence and can help guide future systematic reviews and curriculum development. This protocol has been reported using the Preferred Reporting Items for Systematic Reviews and Meta-analyses extension for systematic review protocols (PRISMA-P) [20] (S1 Appendix).

This review will follow the framework developed by Arksey and O’Malley [21], with refinements proposed by Levac et al. [22] and the Joanna Briggs Institute [23]. The process will involve defining the research questions, identifying relevant studies, selecting eligible studies, charting the data, and synthesizing and reporting the findings. The final scoping review will be reported in accordance with PRISMA-ScR [24].

### Prospective status and study timeline

This manuscript reports a prospective scoping review protocol and does not present findings from the review. At the time of revision, database searching and deduplication have been completed, but formal record screening has not been completed, and data extraction, synthesis, and analysis have not begun. No study-level findings or review results have been generated. Maintaining these stages as prospective ensures that eligibility, extraction, and synthesis procedures are specified without knowledge of the review findings.

The remaining screening, data extraction, and synthesis stages will proceed following a decision on this protocol. Assuming acceptance by August 2026, title and abstract screening is expected to be completed by September 2026, full-text screening by October 2026, data extraction by November 2026, and final synthesis and reporting by December 2026.

### Transparency and reproducibility

To support transparent and reproducible conduct, this protocol was prospectively registered in the Open Science Framework and is reported in accordance with PRISMA-P. The completed review will be reported in accordance with PRISMA-ScR. The Population, Concept, and Context eligibility framework, MEDLINE search strategy, and data extraction framework are provided as Supporting Information. All database-specific search strategies, screening decisions, reasons for exclusion at full-text review, protocol amendments, and deviations from the planned methods will be documented in the completed review and the Open Science Framework record (https://osf.io/jxudv). Screening and data extraction will be undertaken independently by two reviewers, with adjudication by a third reviewer when consensus cannot be reached.

### Ethics statement

Ethical approval and informed consent are not required for this study because it is a scoping review of published and publicly available literature and does not involve human participants, animal subjects, or identifiable personal information.

### Stage 1: Identifying the research question

We used the Population Concept Context framework (PCC), recommended by the Joanna Briggs Institute for scoping reviews to develop our review question [23] (S2 Table).

#### Participants.

This review will consider studies involving physician trainees, including medical students enrolled in undergraduate or graduate entry medical degree programs, such as MD, MBBS, or equivalent entry-to-practice physician qualification pathways, and resident physicians enrolled in structured postgraduate residency or specialty training programs. Medical students may be in either preclinical or clinical phases of training, and resident physicians may be from any specialty or postgraduate year.

Studies will be eligible if they report empirical data related to telemedicine teaching, learning, or assessment involving these groups. Studies including both medical students and residents will also be considered, with data extracted separately when group specific findings are reported. Studies focused exclusively on paramedics, nursing students, pharmacy students, allied health students, or other non-physician health professional trainees will be excluded unless data for medical students or resident physicians can be extracted separately.

#### Concept.

This review will consider studies that explore telemedicine-related clinical skills education among medical students and resident physicians across global regions. Telemedicine education will be conceptualized as a set of clinical competencies rather than a mode of educational delivery and will be examined using a prespecified analytic framework encompassing teaching modalities, learning processes, assessment strategies, and contextual influences on curriculum design and implementation.

Teaching modalities will be defined as structured instructional approaches used to develop telemedicine-related clinical skills. These may include didactic instruction, simulation-based training, standardized patient encounters, supervised clinical telemedicine encounters, role-play, asynchronous modules, and hybrid or longitudinal curricula designed to support skill acquisition.

Learning processes will be defined as learner engagement with telemedicine-related clinical competencies, including communication skills, virtual physical examination techniques, clinical reasoning in remote care settings, patient safety and privacy practices, documentation, and interprofessional coordination in telemedicine contexts. Learning outcomes may include self-reported confidence, perceived preparedness, skill acquisition, and integration of telemedicine competencies into clinical practice.

Assessment strategies will be defined as methods used to evaluate telemedicine-related clinical skills and competencies. These may include formative or summative assessments, direct observation, OSCEs, checklist-based evaluations, reflective exercises, self-assessment measures, faculty evaluations, or competency-based milestones related to telemedicine practice.

Contextual and implementation factors will encompass institutional, regional, cultural, regulatory, and health system influences shaping telemedicine education. These may include resource availability, technological infrastructure, accreditation or licensing requirements, reimbursement policies, legal and ethical considerations, faculty training, and curricular integration within broader medical education programs.

Reported outcomes and implications will include authors’ descriptions of curricular effectiveness, feasibility, acceptability, perceived gaps or unmet educational needs, and recommendations for future curriculum development or policy. Where reported, learner- and program-level outcomes, as well as limitations and challenges in telemedicine clinical skills training, will be charted and synthesized.

#### Context.

This review will consider studies examining telemedicine-related clinical skills education for medical students and resident physicians across global regions. Studies will be eligible if they are conducted within any geographic region or if multi-country analyses report disaggregated data for at least one defined region or country. Telemedicine training may occur in any educational or clinical setting, including academic medical centers, teaching hospitals, community-based training sites, ambulatory clinics, or virtual clinical environments.

No restrictions will be placed on health system structure, regulatory or accreditation frameworks, technological infrastructure, or stage of telemedicine adoption. Cultural, linguistic, and sociocultural factors influencing communication, patient–clinician interaction, and professional norms in telemedicine practice, as well as geographic considerations such as urban–rural settings and resource availability, will be considered when reported.

### Stage 2: Identifying relevant studies

#### Types of sources.

This scoping review will include peer-reviewed empirical and descriptive studies examining telemedicine education for medical students and resident physicians. Eligible study designs will include quantitative, qualitative, mixed methods, interventional, observational, program evaluation, and descriptive curriculum reports that provide sufficient detail on teaching, learning, assessment, or implementation.

Systematic reviews and scoping reviews will not be included as evidence sources in the final map but their reference lists will be screened to identify eligible primary studies. Narrative reviews, editorials, opinion pieces, expert perspectives, conference abstracts without full-text, and documents without sufficient primary data or detailed curriculum description will be excluded.

Studies focused solely on patient outcomes without an educational component will be excluded. Studies involving mixed trainee populations will be included only when data for medical students or resident physicians can be extracted separately, or when physician trainees clearly represent the target educational population.

#### Search strategy.

The search strategy was designed to identify studies examining telemedicine education for medical trainees, including medical students and resident physicians, with a focus on teaching, learning, assessment, and implementation across global settings. An initial limited search of MEDLINE and relevant evidence-synthesis sources was undertaken to identify key articles and refine the search concepts. Text words in the titles and abstracts of relevant records, together with database-specific index terms, were used to develop the initial search strategy presented in S3 Table. This strategy was reviewed by an information specialist, piloted against a set of sentinel studies known to the review team to assess its sensitivity, and subsequently translated and adapted for each database using database-specific subject headings and syntax.

The initial searches were conducted in MEDLINE via PubMed, Embase via Ovid, the Cochrane Central Register of Controlled Trials, ERIC via ProQuest, Web of Science, and CINAHL. The searches covered records published from January 1, 2015, to the date on which each database was searched. The reference lists of all included full-text articles and relevant reviews will also be screened to identify additional eligible studies. A final update search will be conducted before data extraction is completed to identify newly published studies. For consistency in comparative analysis, included studies will be classified by country and grouped using a predefined regional framework and, where feasible, country income category. The selected framework will be applied consistently across all included studies and reported in the final manuscript.

To ensure feasibility, consistency, and reliability of study selection and data extraction, the review will be limited to studies with full-text published in English. This decision reflects the need for detailed extraction of curricular content, teaching approaches, assessment strategies, implementation factors, and authors’ interpretations, which would require formal translation procedures and bilingual verification if non-English full-texts were included. Such procedures are beyond the predefined scope and available resources of the present protocol. Conference abstracts without full-text and gray literature will also be excluded. The potential effect of this language restriction on geographic representativeness will be considered when interpreting the findings.

### Stage 3: Study selection

All records retrieved from the searches will be exported to Zotero (Corporation for Digital Scholarship, Vienna, VA, USA) for reference management and deduplication, then imported into Covidence for screening [25]. Study selection will be conducted in two stages: title and abstract screening, followed by full-text screening.

Before formal screening begins, the review team will pilot the eligibility criteria on a sample of records to ensure shared understanding and consistent application of the inclusion and exclusion criteria. Two reviewers will then independently screen titles and abstracts against the predefined eligibility criteria. Records considered potentially relevant by either reviewer will proceed to full-text review. Two reviewers will independently assess full-text articles for eligibility, and reasons for exclusion at the full-text stage will be recorded. Disagreements at any stage will be resolved through discussion. If consensus cannot be reached, a third reviewer will adjudicate. When full-texts are not readily accessible, reasonable efforts will be made to obtain them through institutional library access or contact with the corresponding author before exclusion. The final study selection process will be presented in a PRISMA-ScR flow diagram.

### Stage 4: Charting the data

Data will be extracted from included studies using a structured extraction form developed a priori and aligned with the review objectives (S4 Table). The form will be piloted on a subset of included studies to confirm clarity, completeness, and consistent interpretation, and refined as needed. Any modifications to the extraction form will be documented and reported in the final review.

Two reviewers will independently extract data from all included studies. Disagreements will be resolved through discussion, with consultation from a third reviewer when necessary. Extracted data will include study characteristics, participant characteristics, curriculum characteristics, teaching and learning modalities, assessment approaches, implementation context, reported outcomes, authors’ interpretations, recommendations, and limitations. When multiple reports describe the same curriculum or study population, these reports will be linked and treated as a single evidence unit where appropriate, with relevant information extracted from all associated publications to avoid double counting.

### Stage 5: Collating, summarizing and reporting the results

Extracted data will be synthesized descriptively in both tabular and narrative formats. Study characteristics will be summarized by year of publication, country, region, learner level, specialty, study design, and educational setting. Curriculum characteristics will be mapped by content, teaching modality, learning activity, assessment approach, duration, delivery format, and degree of curricular integration.

Findings will be organized primarily according to the review’s a priori analytic framework: teaching modalities, learning processes, assessment strategies, and contextual or implementation factors. Within each domain, studies will be compared across learner level and geographic region. Where feasible, regional comparisons will also consider country income category and resource context.

Quantitative findings will primarily be summarized using counts, frequencies, and distributions, as substantial heterogeneity in study design, curriculum structure, learner population, and outcome reporting is anticipated. However, where a subset of studies demonstrates sufficient similarity in study design, curriculum structure, learner group, intervention characteristics, and outcome reporting, comparable quantitative findings may be grouped and, where appropriate, pooled descriptively. Formal statistical pooling will only be considered if data are sufficiently homogeneous and reported using compatible outcome metrics. Otherwise, findings will be synthesized narratively and presented in tables. Qualitative and narrative findings will be synthesized using descriptive content analysis to identify recurring themes related to barriers, facilitators, educational gaps, learner attitudes, perceived preparedness, feasibility, and acceptability. Authors’ interpretations and recommendations will be charted alongside reported outcomes to support a comprehensive understanding of how telemedicine curricula are designed, implemented, and evaluated globally.

### Stage 6: Consultation

Consistent with the recommendation of Levac et al. [22], consultation will be incorporated as an optional but valuable component of this scoping review. Following preliminary synthesis of the included studies, the findings may be shared with medical educators, clinical faculty, and physicians involved in undergraduate and postgraduate training to support interpretation of the results and enhance their relevance to educational practice. This consultation process will help contextualize the emerging findings, identify their implications for curriculum design and implementation, explore practical strategies for dissemination, and highlight priorities for future research. Through engagement with educators and clinicians, the review will benefit from perspectives grounded in real world teaching and clinical settings, particularly regarding the development, assessment, and integration of telemedicine competencies in medical training.

## Limitations

Although every effort will be made to identify all relevant studies, it is possible that some eligible literature may not be captured by the search strategy. In addition, the review will be limited to studies with full-text published in English. This restriction may introduce language and geographic publication bias, particularly because telemedicine education may be reported in national languages in regions such as Latin America, Eastern Europe, Asia, Africa, and other non-English-speaking settings. As a result, the review may overrepresent evidence from English-language and highly indexed publication contexts, and the findings should be interpreted as a map of English-language evidence across global regions rather than a complete representation of all telemedicine education worldwide. This limitation will be explicitly considered in the interpretation of regional patterns and evidence gaps.

The review is also restricted to medical students and resident physicians. This focused scope is intended to ensure that the findings are directly relevant to physician training and to the development of medical curricula, assessment approaches, and educational policy. However, this may limit the applicability of the findings to other health professions and to interprofessional education contexts, which may warrant separate investigation in future research. Finally, substantial heterogeneity is anticipated across study designs, learner groups, curricular models, outcome measures, and implementation settings. As a result, the review will emphasize descriptive mapping and narrative synthesis rather than direct comparison of effectiveness across studies.

## Supporting information

S1 Appendix PRISMA-P checklist. (DOCX)

S2 Table Population, concept, and context eligibility framework. (DOCX)

S3 Table MEDLINE search strategy. (DOCX)

S4 Table Data extraction framework. (DOCX)

## References

[1] Spaulding EM, Fang M, Commodore-Mensah Y, Himmelfarb CR, Martin SS, Coresh J. Prevalence and disparities in telehealth use among US adults following the COVID-19 pandemic: national cross-sectional survey. J Med Internet Res. 2024;26:e52124. doi: 10.2196/52124 PMC11127137 38728070

[2] Wosik J, Fudim M, Cameron B, Gellad ZF, Cho A, Phinney D, et al. Telehealth transformation: COVID-19 and the rise of virtual care. J Am Med Inform Assoc. 2020;27(6):957–62. doi: 10.1093/jamia/ocaa067 32311034 PMC7188147

[3] Pylypchuk Y, Barker W. Use of telemedicine among office-based physicians, 2021. ASTP Health IT Data Brief. Washington (DC): Office of the Assistant Secretary for Technology Policy; 2023. 39475515

[4] Omboni S, Padwal RS, Alessa T, Benczúr B, Green BB, Hubbard I, et al. The worldwide impact of telemedicine during COVID-19: current evidence and recommendations for the future. Connect Health. 2022;1:7–35. doi: 10.20517/ch.2021.03 35233563 PMC7612439

[5] Shaver J. The state of telehealth before and after the COVID-19 pandemic. Prim Care. 2022;49:517–30. doi: 10.1016/j.pop.2022.04.002 PMC9035352 36357058

[6] Kane CK. Patient-facing telehealth: use is higher than pre-pandemic but with great variation across physician specialties. 2025. Accessed 2026 March 20. https://www.ama-assn.org/system/files/2024-prp-telehealth.pdf

[7] Jiang Q, Deng Y, Perle J, Zheng W, Chandran D, Chen J, et al. Education and training of telemental health providers: a systematic review. Front Public Health. 2024;12:1385532. doi: 10.3389/fpubh.2024.1385532 38841687 PMC11152158

[8] Khullar D, Mullangi S, Yu J, Weems K, Shipman SA, Caulfield M, et al. The state of telehealth education at U.S. medical schools. Healthc (Amst). 2021;9(2):100522. doi: 10.1016/j.hjdsi.2021.100522 33548854

[9] Cruz-Panesso I, Tanoubi I, Drolet P. Telehealth competencies: training physicians for a new reality?. Healthcare. 2023;12:93. doi: 10.3390/healthcare12010093 PMC10779292 38200999

[10] Wamsley M, Cornejo L, Kryzhanovskaya I, Lin BW, Sullivan J, Yoder J, et al. Best practices for integrating medical students into telehealth visits. JMIR Med Educ. 2021;7(2):e27877. doi: 10.2196/27877 33881407 PMC8100882

[11] Setyonugroho W, Kennedy KM, Kropmans TJB. Reliability and validity of OSCE checklists used to assess the communication skills of undergraduate medical students: A systematic review. Patient Educ Couns. 2015;S0738-3991(15)00277-3. doi: 10.1016/j.pec.2015.06.004 26149966

[12] Sartori DJ, Hayes RW, Horlick M, Adams JG, Zabar SR. The TeleHealth OSCE: preparing trainees to use telemedicine as a tool for transitions of care. J Grad Med Educ. 2020;12(6):764–8. doi: 10.4300/JGME-D-20-00039.1 33391602 PMC7771608

[13] Noronha C, Lo MC, Nikiforova T, Jones D, Nandiwada DR, Leung TI, et al. Telehealth competencies in medical education: new frontiers in faculty development and learner assessments. J Gen Intern Med. 2022;37(12):3168–73. doi: 10.1007/s11606-022-07564-8 35474505 PMC9040701

[14] Bajra R, Srinivasan M, Torres EC, Rydel T, Schillinger E. Training future clinicians in telehealth competencies: outcomes of a telehealth curriculum and teleOSCEs at an academic medical center. Front Med (Lausanne). 2023;10:1222181. doi: 10.3389/fmed.2023.1222181 37849494 PMC10577422

[15] Ampadu JV, Perez A. Telehealth virtual simulation for community health nursing students. Clin Simul Nurs. 2023;83:101442. doi: 10.1016/j.ecns.2023.101442

[16] Kumra T, McGuire M, Stein A, Pahwa AK. Telemedicine clinical skills needs assessment in early medical students. Fam Med. 2022;54(4):294–7. doi: 10.22454/FamMed.2022.904873 35421245

[17] Rahmah L, Purnomo AF, Alizadeh M, Askari SS, Lutfiana NC, Mundakir M, et al. Are we all singing from the same song sheet? Standardizing terminology used in inter-professional telehealth education and practice: a mixed method study. BMC Med Educ. 2025;25(1):649. doi: 10.1186/s12909-025-07207-6 40325451 PMC12051297

[18] Chike-Harris KE, Durham C, Logan A, Smith G, DuBose-Morris R. Integration of telehealth education into the health care provider curriculum: a review. Telemed J E Health. 2021;27(2):137–49. doi: 10.1089/tmj.2019.0261 32250196

[19] Munn Z, Peters MDJ, Stern C, Tufanaru C, McArthur A, Aromataris E. Systematic review or scoping review? Guidance for authors when choosing between a systematic or scoping review approach. BMC Med Res Methodol. 2018;18(1):143. doi: 10.1186/s12874-018-0611-x 30453902 PMC6245623

[20] Moher D, Shamseer L, Clarke M, Ghersi D, Liberati A, Petticrew M, et al. Preferred reporting items for systematic review and meta-analysis protocols (PRISMA-P) 2015 statement. Syst Rev. 2015;4(1):1. doi: 10.1186/2046-4053-4-1 25554246 PMC4320440

[21] Arksey H, O’Malley L. Scoping studies: towards a methodological framework. Int J Soc Res Methodol. 2005;8(1):19–32. doi: 10.1080/1364557032000119616

[22] Levac D, Colquhoun H, O’Brien KK. Scoping studies: advancing the methodology. Implement Sci. 2010;5:69. doi: 10.1186/1748-5908-5-69 20854677 PMC2954944

[23] Peters MDJ, Godfrey CM, Khalil H, McInerney P, Parker D, Soares CB. Guidance for conducting systematic scoping reviews. Int J Evid Based Healthc. 2015;13(3):141–6. doi: 10.1097/XEB.0000000000000050 26134548

[24] Tricco AC, Lillie E, Zarin W, O’Brien KK, Colquhoun H, Levac D, et al. PRISMA Extension for Scoping Reviews (PRISMA-ScR): checklist and explanation. Ann Intern Med. 2018;169(7):467–73. doi: 10.7326/M18-0850 30178033

[25] Covidence systematic review software. Accessed 2026 March 20. https://www.covidence.org/

