# Supplementary material for: Telemedicine education for medical students and residents worldwide: A scoping review protocol
Source: PLoS One. 2026 Oct 1;21(10):e0359776. doi: 10.1371/journal.pone.0359776 (PMC13630243; doi:10.1371/journal.pone.0359776)
Supplement: Supplementary file 2 [file pone.0359776.s002.docx]

### **S2 Table: Population, Concept, and Context Eligibility Framework**

| **Inclusion Criteria** | **Exclusion Criteria** |
| --- | --- |
| A. **Population**: Medical students enrolled in undergraduate or graduate entry physician training programs, including MD, MBBS, or equivalent entry to practice medical degree programs, and resident physicians enrolled in structured postgraduate residency or specialty training programs, regardless of specialty or postgraduate year. B. **Concept**: Telemedicine-related clinical skills education is a key focus, including teaching, learning, or assessment of competencies such as virtual communication, remote clinical reasoning, virtual examination, documentation, digital professionalism, or technology use in clinical care. C. **Context**: Set in any geographic region or educational/clinical setting, including academic medical centers, teaching hospitals, ambulatory clinics, community training sites, or virtual clinical environments. D. **Type of source**: Peer-reviewed empirical and descriptive studies, including quantitative, qualitative, mixed methods, interventional, observational, program evaluation, and descriptive curriculum reports with sufficient detail on teaching, learning, assessment, or implementation. E. **Publication language/date**: Written in English and published between January 1, 2015 and the date of the final search. | A. **Population**: Studies exclusively involving paramedics, nursing students, pharmacy students, allied health students, or other non-physician health professional trainees where data for medical students or resident physicians cannot be extracted separately. B. **Concept**: Telemedicine is not an explicit educational focus; studies reporting only patient outcomes without a trainee education component, or examining general digital literacy or EHR training unrelated to clinical telemedicine skills. C. **Context**: Not set within a medical education program or clinical training context for physician trainees. D. **Type of source**: Systematic reviews and scoping reviews (reference lists screened for primary studies), narrative reviews, editorials, opinion pieces, expert perspectives, conference abstracts without full text, and studies without sufficient primary data or curriculum description. E. **Publication language/date**: Written in a language other than English or published prior to January 1, 2015. |
