# Supplementary material for: Telemedicine education for medical students and residents worldwide: A scoping review protocol
Source: PLoS One. 2026 Oct 1;21(10):e0359776. doi: 10.1371/journal.pone.0359776 (PMC13630243; doi:10.1371/journal.pone.0359776)
Supplement: Supplementary file 3 [file pone.0359776.s003.docx]

**S3 Table: Search Strategy**

| **MEDLINE (via PubMed)**  *Search conducted: January 15^th^, 2026* | | |
| --- | --- | --- |
| **Search** | **Query** | **Records retrieved** |
| #1 Telemedicine block | ("Telemedicine"[Mesh] OR telemedicine[tiab] OR telehealth[tiab] OR teleconsult*[tiab] OR "virtual care"[tiab] OR "virtual visit*"[tiab] OR "remote consultation*"[tiab] OR "digital health"[tiab] OR eHealth[tiab] OR mHealth[tiab] OR "mobile health"[tiab]) | 85,918 |
| #2 Education block | ("Curriculum"[Mesh] OR curriculum[tiab] OR teaching[tiab] OR training[tiab] OR learning[tiab] OR education[tiab] OR instruction[tiab] OR competencies[tiab] OR competency[tiab] OR skills[tiab] OR assessment[tiab] OR evaluation[tiab] OR "Program Evaluation"[Mesh] OR OSCE[tiab] OR "Objective Structured Clinical Examination"[tiab] OR exam*[tiab] OR feedback[tiab]) | 4,205,457 |
| #3 Learner block | ("Students, Medical"[Mesh] OR "Schools, Medical"[Mesh] OR "Internship and Residency"[Mesh] OR medical student*[tiab] OR medical school*[tiab] OR resident*[tiab] OR residency[tiab] OR intern*[tiab] OR trainee*[tiab]) | 912,624 |
| #4 Final set | #1 AND #2 AND #3 | 9,730 |
| Limited to "2015/01/01"[Date - Publication] : "2026/01/15"[Date - Publication]), English | | |
