# Supplementary material for: Telemedicine education for medical students and residents worldwide: A scoping review protocol
Source: PLoS One. 2026 Oct 1;21(10):e0359776. doi: 10.1371/journal.pone.0359776 (PMC13630243; doi:10.1371/journal.pone.0359776)
Supplement: Supplementary file 4 [file pone.0359776.s004.docx]

### **S4 Table: Data Extraction Framework**

| **Domain** | **Variable** | **What to record / coding instructions** |
| --- | --- | --- |
| Study identification | Study ID | Assign a unique ID for each included study |
|  | Full citation | First author, year, title, journal |
|  | Year of publication | Year published |
|  | Country | Country where the study was conducted |
|  | Region | Code by predefined region for analysis, for example North America, Europe, East Asia, Southeast Asia, South Asia, Sub Saharan Africa, Latin America, Middle East and North Africa, Oceania |
|  | Multi country study | Yes or no. If yes, list all countries |
| Study design and methods | Study design | Quantitative, qualitative, mixed methods, program evaluation, descriptive curriculum report, interventional, observational, or other |
|  | Study aim | Main aim of the paper, for example curriculum development, implementation, evaluation, learner perceptions, competency assessment |
|  | Data source | Surveys, interviews, focus groups, curriculum documents, assessments, institutional records, or multiple |
|  | Study setting | Medical school, teaching hospital, residency program, ambulatory clinic, virtual setting, community site, or multiple |
|  | Time frame | Year or years of curriculum delivery, implementation, or evaluation |
|  | Pandemic context | Pre COVID 19, during COVID 19, post COVID 19, or unclear |
| Participants | Learner group | Medical students, residents, or both |
|  | Level of training | Preclinical student, clinical student, postgraduate year, mixed, or unclear |
|  | Specialty | General undergraduate medical education, internal medicine, family medicine, pediatrics, surgery, psychiatry, or other |
|  | Sample size | Number of learners included. If faculty are also included, record separately |
|  | Other stakeholders | Faculty, program directors, standardized patients, patients, or none |
|  | Prior telemedicine exposure | Report if stated, for example none, limited, prior experience, not reported |
| Curriculum characteristics | Curriculum title or name | Name of curriculum or program if reported |
|  | Curriculum purpose | Orientation, skill building, clinical exposure, assessment, remediation, longitudinal training, or multiple |
|  | Curriculum integration | Standalone course, embedded in clerkship, embedded in residency, longitudinal, elective, or hybrid |
|  | Duration | Number of sessions, weeks, months, or total hours if reported |
|  | Frequency | One time, repeated sessions, longitudinal, or unclear |
|  | Delivery format | In person, virtual, hybrid, asynchronous, synchronous, or mixed |
|  | Telemedicine context | Teleconsultation, virtual follow up, remote triage, telemonitoring, mixed, or unclear |
| Curriculum content | Content domains taught | Communication, virtual physical examination, clinical reasoning, documentation, digital professionalism, ethics, legal issues, privacy/confidentiality, technology use, patient safety, health equity/access, interprofessional practice, or other |
| Teaching and learning approaches | Teaching modality | Lecture, workshop, simulation, standardized patient, case based learning, supervised clinical encounter, e learning module, peer teaching, or other |
|  | Learning activities | Observation, role play, practice encounter, feedback session, reflective exercise, case discussion, or other |
|  | Faculty development component | Yes, no, or not reported |
| Assessment | Assessment used | None reported, formative assessment, summative assessment, OSCE, checklist, direct observation, knowledge test, self assessment, faculty evaluation, reflective assignment, or other |
|  | Outcome measures | Confidence, satisfaction, knowledge score, skill performance, preparedness, acceptability, feasibility, behavior change, or other |
|  | Timing of assessment | Immediate post curriculum, short term follow up, long term follow up, multiple time points, or not reported |
| Results | Main findings | Concise summary of principal findings relevant to the review questions |
|  | Learner outcomes | Knowledge, skills, attitudes, confidence, preparedness, performance, or mixed |
|  | Implementation findings | Barriers, facilitators, feasibility issues, resource needs, faculty issues, technological issues, regulatory issues, or other |
|  | Regional or contextual influences | Any reported influence of country, region, resources, culture, language, urban rural setting, or health system |
|  | Reported gaps | Unmet needs, missing competencies, limited evaluation, lack of faculty preparation, lack of standardization, or other |
| Interpretation | Authors’ conclusions | Authors’ main interpretation of the curriculum or intervention |
|  | Recommendations | Recommendations for curriculum development, policy, faculty development, or research |
| Quality and completeness of reporting | Limitations reported by authors | Record the study limitations as stated by the authors |
|  | Missing information | Note important missing details, for example duration not reported, assessment unclear, sample characteristics incomplete |
